# Computational Link Between Motivational Factors and Cognitive Deficits in Depression

**DOI:** 10.1007/s12559-026-10635-1

**Published:** 2026-11-03

**Authors:** Aleks Stolicyn, Liana Romaniuk, Stephen M. Lawrie, Peggy Seriès

**Affiliations:** 1 Division of Psychiatry, Centre for Clinical Brain Sciences, University of Edinburgh, Chancellor’s Building, 49 Little France Crescent, Edinburgh BioQuarter, Edinburgh, EH16 4SB UK; 2 Institute for Machine Learning, School of Informatics, University of Edinburgh, 10 Crichton Street, Edinburgh, EH8 9AB UK; 3 School of Health and Wellbeing, University of Glasgow, Clarice Pears Building, 90 Byres Road, Glasgow, G12 8TB UK

**Keywords:** Depression, Cognition, Cognitive control, Motivation, Computational modelling

## Abstract

Cognitive deficits are a common symptom of depression and contribute significantly to the disabling effects of the disorder. Experimentally, they are observed as increased reaction times, increased error rates, and deficient performance adaptation after making errors or receiving adverse feedback, in multiple cognitive paradigms. In the current study we aimed to address the cause of these cognitive deficits from a theoretical perspective. We constructed computational models of optimal resource allocation in two cognitive tasks – Delayed Match to Sample (DMS), and Eriksen Flanker (EF). The models explicitly link performance feedback values and beliefs about task controllability with measures of cognitive performance including accuracy, reaction times, and post-error improvement in accuracy (PIA). We then introduced depression-related motivational changes – altered control belief and feedback values (representing learned helplessness, anhedonic valuation and negative bias) – to see if these factors can account for deficits in cognitive performance. In the DMS task, altered control belief and lower valuation of correct performance accounted for decreased accuracy and decreased PIA. In the EF task, these two factors could explain increased response times, decreased accuracy and decreased error-related negativity (ERN) signal magnitude. Increased valuation of adverse feedback, on the other hand, was linked to increased EF accuracy and increased ERN signal magnitude. Furthermore, in the EF task, different combinations of depression-related motivational factors led to different patterns of cognitive performance, which could offer a basis for stratification. Our study offers a novel perspective on how cognitive deficits in depression can be viewed as consequences of altered motivational factors. The developed models provide an explicit computational and algorithmic bridge between the commonly observed cognitive deficits (increased reaction times, decreased performance accuracy, worse post-error adaptation) and factors such as learned helplessness and anhedonic valuation.

**Supplementary Information:**

The online version contains supplementary material available at https://doi.org/10.1007/s12559-026-10635-1.

## Introduction

Depression (major depressive disorder) is one of the most common psychiatric conditions with an average lifetime prevalence of between 10% and 15%, and 12-month prevalence of around 6% in the general population [1–3]. The two core diagnostic criteria of depression are low mood and loss of interest in daily activities (anhedonia), typically accompanied by other cognitive and somatic symptoms, for a period of at least two weeks [4, 5]. Depression has been estimated to cause substantial societal and economic burdens, likely at least in part due to changes in cognition [6–9].

One significant clinical feature of depression is the presence of cognitive deficits. Reduced concentration and cognitive ability is considered part of the symptomatology [4, 5, 10, 11]. Pronounced deficits have also been observed in executive function, covering the domains of attention and working memory [12–19]. *Executive function* here refers to a set of fundamental cognitive abilities that underpin goal-directed behaviour – these include working memory and attention, and others such as planning and task shifting [20, 21]. *Working memory* refers to the ability to temporarily store, retrieve and update information necessary for completion of simpler or more complex tasks, such as reasoning or adhering to instructions [20, 22]. *Attention* refers to the ability to selectively process information pertinent to the current task while ignoring or discarding distractions [23]. *Working memory* and *attention* work together and comprise essential cognitive functions for daily life. Multiple reviews indicate that working memory is impaired in depression [12–16, 18, 24], which is evidenced by poorer performance at domain-specific cognitive tasks such as the N-back task and the Delayed Match to Sample (DMS) task [12, 17]. Review evidence similarly indicates diminished attention [12, 14–16, 24, 25], manifesting as poorer performance at tasks such as the Stroop task, Trail-making test and the Eriksen Flanker task [26–28]. Depression-related deficits at these tasks are typically observed as increased reaction times (e.g., at Stroop and Eriksen Flanker tasks [13, 26–28]), and as decreased accuracies (percentages of trials with correct responses, e.g., at the N-back and DMS tasks [12, 13, 17, 29]).

One further behavioural effect representative of cognitive deficits in depression is the diminished post-error improvement in accuracy (PIA). Post-error improvement in accuracy is not universal but is often observed in healthy participants across different cognitive paradigms when improvement in performance is possible [30, 31]. In depression, diminished PIA effect has been termed ‘catastrophic response to failure’ and has been consistently found in experimental paradigms with trial-by-trial feedback, including the DMS and the Tower of London tasks [32–44]. In paradigms without explicit feedback, the evidence is mixed: the diminished PIA effect has been found in some studies [45–47], but not in others [28, 48, 49]. This is likely because errors are perceived differently and less consistently in tasks without trial-by-trial feedback, with depression affecting error reactions in some such tasks more than in others.

From the neurophysiological perspective, two event-related potentials occur in the brain when the experimental participant makes an error (as measured with electroencephalography) – error-related negativity (ERN) and feedback-related negativity (FRN). Briefly, both ERN and FRN are negatively-going frontocentral signal deflections which occur approximately 80–120 ms after commission of errors (ERN), or 250–300 ms after presentation of negative feedback (FRN) [50–52]. These signals are considered to represent the neural processes behind cognitive adaptation to errors and feedback [50, 53, 54]. The majority of the evidence indicates that the FRN signal is decreased in depression, potentially linked to altered reward processing [55–58], however this is inconsistent and increases have been reported in some studies [57, 59–62]. Regarding the ERN signal, the evidence is mixed, with both increases and decreases reported in depression [58, 63–65]. This contrasts with anxiety disorders, where robust increases in ERN have been found [64–68]. One likely reason for the inconsistent findings of the ERN / FRN is the heterogeneity of depression [69, 70] – increases or decreases in these signals could be related to the predominant symptoms such as anhedonia, low mood, or increased threat sensitivity [63, 64].

From the perspective of motivation, depression can be characterised by three core changes: decreased estimates of behavioural control, decreased reward sensitivity, and negatively biased affective cognition. Decreased behavioural control is the core tenet of the *learned helplessness* theory of depression. The theory suggests that when animals or humans are continuously exposed to uncontrollable and undesirable events, they learn to adopt passive behavioural coping strategies, characterised by apathy and lack of exploration, which is considered to represent core depression symptoms [71–73]. Decreased reward sensitivity, also interpreted as lower valuation of rewards, is closely related to the symptom of anhedonia, and has been shown in multiple computational modelling and other studies of depression [74–76]. Finally, negative affective bias is the propensity to attend to and remember negative material. Negative bias is the core feature of cognitive theories of depression, which suggest that the bias is triggered by adverse environmental circumstances and leads to symptoms such as low mood [77–79]. One interpretation of the negative bias from a computational perspective is in abnormally high valuation of adverse outcomes and, consequently, cues for such outcomes (i.e., negative material) [76, 80].

Several theoretical accounts have recently linked motivational factors such as outcome valuation and controllability to cognitive performance. One of these accounts is the Expected Value of Control (EVC) theory, which suggests that cognitive control (effort) is deployed on any task in a way so as to maximise the trade-off between the expected rewards and the costs of effort [81–83]. In the EVC account, cognitive performance directly depends on the amount of cognitive effort deployed, which is in turn based on the estimated probabilities and values of outcomes (e.g., values of the positive and negative feedback), and on the cognitive effort costs. An advantage of the EVC theory is that it is a principled normative account of optimal cognitive effort allocation that can be put in precise computational terms. The EVC theory has recently been suggested as a framework capable of causally linking motivational aspects of depression, such as decreased reward sensitivity and perceived lack of behavioural control, to deficits in cognitive control [84]. As such, the EVC framework is uniquely well-placed for (1) development of new computational models that link motivational factors to performance at different cognitive tasks, and (2) investigation, from a normative theoretical perspective, of how depression-related motivational changes are linked to task performance.

In the current study we aimed to provide a theoretical (normative) explanation of depression-related performance differences in two cognitive tasks – DMS and Eriksen Flanker (EF). In depression, accuracy is decreased in the DMS task [12, 33, 35, 85–91], and some evidence indicates that there is decreased PIA effect [33–35]. In the EF task, reaction times are increased, especially at incongruent trials [28, 48, 92–96], while accuracy can be either decreased [92, 97], increased [28, 98], or unchanged [48, 93–96, 99–101]. Furthermore, some studies found increased ERN signal in the task [93, 94, 98], while others found an opposite effect [92, 99, 100]. These discrepant results are likely due to the heterogeneity of depression [69, 70], however it remains an open question as to how different depression subtypes map to different patterns of changes in performance.

The DMS and EF tasks were chosen because they meet the following four criteria: (1) these two tasks respectively index working memory and attention – two cognitive domains important for everyday function but impaired in depression; (2) existing evidence indicates there are at least some depression-related impairments at these tasks, most likely reflecting deficits in working memory and attention; (3) these two tasks are suitable for normative computational modelling with the EVC framework, because the quantity of cognitive control (effort) and the reward-effort trade-off can be mathematically formalised – yet this, to our knowledge, has not been done before; and (4) sufficient experimental data is available in the published reports to constrain the parameters of the computational models [28, 33, 35] (supplementary sections S1.8 and S2.7). The DMS and EF tasks have previously been considered from the mechanistic perspective in neural network and drift-diffusion models [28, 102]; we here complement this perspective by showing how differences in normative (motivational) aspects, rather than mechanisms, also factor into performance at these tasks.

We aimed to address a key unresolved issue – what causes the depression-related performance deficits? To date this question has only been addressed partly from a mechanistic neurobiological perspective [11, 18, 103], but not from a normative one [84]. Some of the cognitive mechanisms potentially responsible for the performance deficits could include reduced effort allocation in cognitively demanding tasks [104, 105], which in turn may be linked to anhedonic valuation [75, 76, 104, 106], beliefs of helplessness [71], or disruption from task-irrelevant emotional processes such as rumination [9, 107]. In the DMS task, reduced effort could adversely affect the sample encoding and maintenance, making it more difficult to identify the matching sample at the selection stage. In the EF task, reduced effort could translate to poorer focus on the target stimulus, and thus higher attentional interference from the flankers. We here aimed to offer an algorithmic and normative account that formally links the motivational aspects of depression – i.e. reduced effort allocation, anhedonic valuation, belief of helplessness – to deficits in cognitive performance. A distinct advantage of a normative explanation is that it takes into account the core affective features of depression [84], beyond just brain mechanisms, which in turn can offer a novel direction for identifying treatments that are not solely focused on neurobiology [108].

We here did not collect or analyse new experimental data, but addressed the question from a theoretical perspective, aiming to consider previously published data [28, 33–35]. We constructed new formal computational models, consistent with the EVC theory, that link depression-relevant motivational factors to performance at the DMS and EF tasks. We then simulated depression-related motivational changes – decreased controllability estimate, decreased reward sensitivity, and increased negative valuation – to see if these factors can causally explain the commonly observed performance deficits (decreased accuracy at the DMS task and increased reaction times at the EF task [28, 33–35]). Finally, we aimed to check if different depression-related motivational factors can lead to different patterns of performance at the EF task, which could explain the discrepant results in the previous studies.

## Methods and Models

### General Model Framework

Our general framework for cognitive tasks is based on the EVC theory, with an addition of an explicit component to model belief of control – i.e. the (simulated) participant’s estimate of how likely their efforts are to affect the environment. Briefly, at each trial of a cognitive task, we assume that participants combine their knowledge of the task, values of outcomes (i.e., error and correct response feedback), costs of cognitive resources (cognitive effort), and their belief about task controllability, to estimate how much effort is optimal to deploy for completing the trial. Consistently with the EVC theory, optimal amount of effort is the one that maximises the trade-off between the expected outcome value and the costs of expended effort, which we here also term *utility* (* U*):

1 $$U\left(f\right)=\underbrace{V_c\cdot\:P_{cw}\left(f,{\alpha\:}_c\right)+V_e\cdot\:\left(1-P_{cw}\left(f,{\alpha\:}_c\right)\right)}_{expected\:outcome\:value}-\underbrace{{\eta\:}_c\cdot\:f}_{cognitive\:costs}$$

2 $$\:{f}_{opt}=\underset{f}{argmax}\left(U\left(f\right)\right)$$

In the above equation * f* represents the amount of cognitive resources deployed at the trial, $\:{V}_{c}$ and $\:{V}_{e}$ are respectively values of correct and error outcomes, $\:{\eta\:}_{c}$ is the cost of cognitive effort, and $\:{P}_{cw}$ is the participant’s estimated probability of positive outcome (correct response), which depends on the participant’s knowledge of the task, the deployed cognitive effort and the participant’s belief of control $\:{\alpha\:}_{c}$. We define belief of control ($\:{\alpha\:}_{c}$) as the participant’s estimate of the probability that different levels of effort (* f*) lead to different trial results (success probabilities), which is consistent with the second notion of behavioural control in Huys & Dayan (2009) [109]. This notion is relevant here because it can capture the concept of *learned helplessness* in the environments where all outcomes (positive and negative feedback) do occur, but may be perceived or believed to be not dependent on the action (i.e. exerted effort) – which applies to forced-choice tasks such as the DMS and EF. $\:{f}_{opt}$ is the optimal amount of cognitive effort which maximises utility, and * argmax* is the *argument of the maximum* function – i.e., the function which identifies the target function’s ($\:U\left(f\right)$ in our case) input argument of the maximal function value. Note that the allocated cognitive resources * f* are used directly to complete the task trial (i.e. to memorise or focus on the relevant stimuli – supplementary sections S1.1 and S2.1), and thus * f* directly influences the probability of correct response at controllable tasks, however this may not always be immediately perceived by the participant if they believe that their control is limited.

After defining the optimal amount of cognitive resources and completing a single trial, participant receives feedback * F* which they use to update their knowledge of the task or its difficulty. This updated knowledge is subsequently used for defining optimal cognitive resources at the next trial, which serves as the basis for performance adaptation and the PIA effect. Dependent on the cognitive paradigm, these updates can take the form of full statistical learning or a heuristic process of adapting to the task demands drawing on the recent trials.

Prominent theoretical accounts of the FRN and ERN potentials suggest that the magnitude of these signals reflects *valued prediction error*, that is the difference between the expected outcome (trial result) value and the actually received outcome value [110–113]. Drawing on this previous work, we have taken valued prediction errors as a proxy for the magnitude of the FRN (ERN) signals in our models, and computed them as follows:

3 $${FRN} = \underbrace{PEv}_{\substack{{\mathrm{valued}} \\ {\mathrm{prediction}} \\ {\mathrm{error}}}} = \underbrace{P_{cw}(f_{opt}) \cdot V_c + (1 - P_{cw}(f_{opt})) \cdot V_e}_{{\text{expected outcome value}}} - \underbrace{(F \cdot V_c + (1 - F) \cdot V_e)}_{{\text{actual outcome value}}}$$

**Fig. 1:**
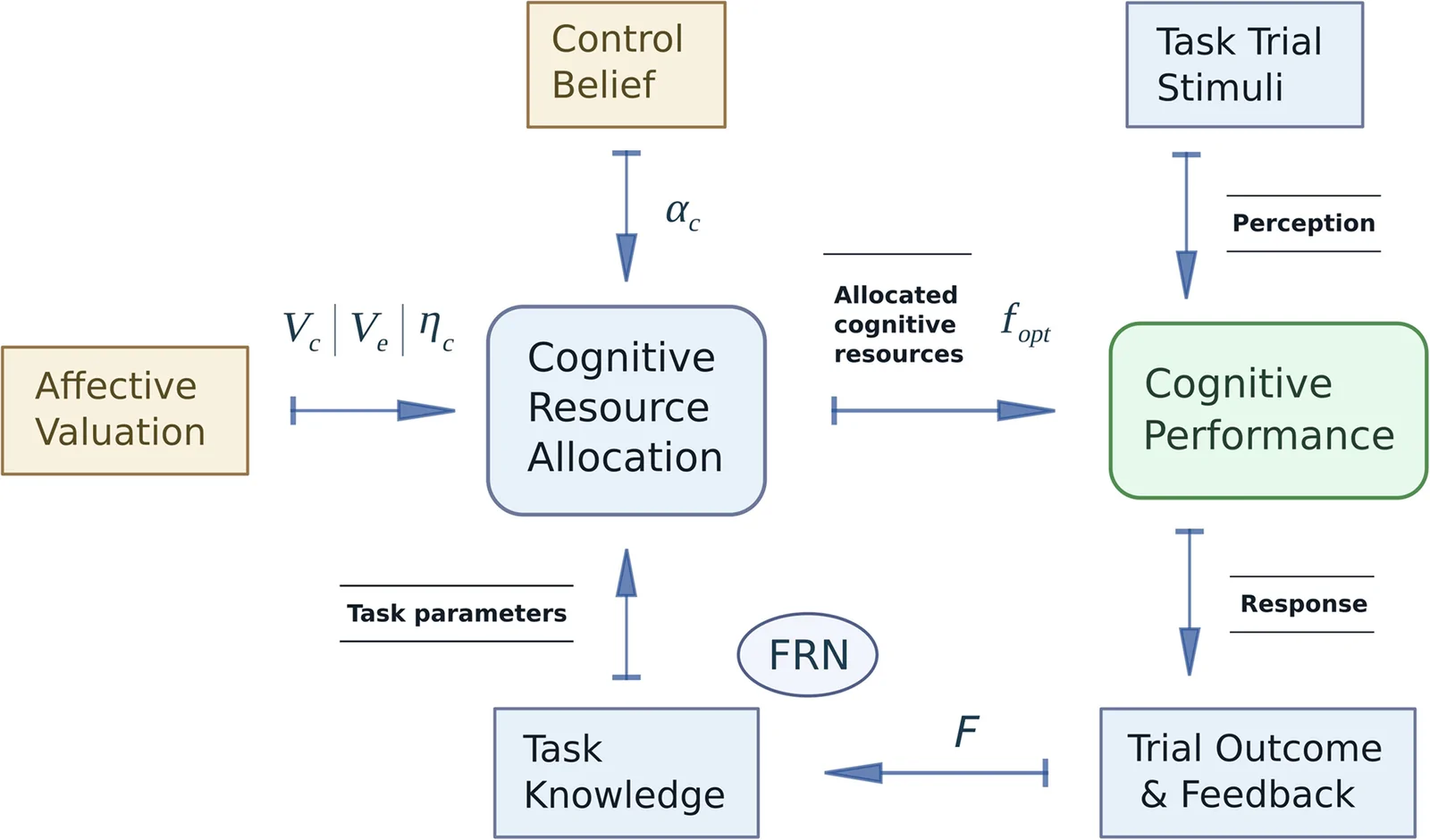
General modelling framework to link motivational factors to cognitive performance. Motivational factors (belief of control, valuations of outcomes and cognitive resources) are combined with the knowledge of the task to define the optimal amount of cognitive resources to deploy at the task trial. Cognitive resources are then deployed, and trial feedback is received. Feedback is used to update the participant’s knowledge of the task or task difficulty, which are used at the next trial. In the current, study we aimed to investigate how depression-related changes in motivational factors (highlighted in yellow) are linked to cognitive performance (highlighted in green)

Figure 1 illustrates the general framework within which we further adapted the computational models for the DMS and EF tasks. We below describe our formalisation of these two tasks in more detail.

### Delayed Match to Sample Model

Briefly, each trial of the DMS task starts with presentation of an abstract visual pattern (the sample), consisting of a number of features. In typical implementations of the task, features are the colours and shapes of the separate quadrants of a rectangular pattern [35, 90, 114]. The sample then disappears and after a short delay (typically 4 to 12 s) a selection of patterns appears (4 patterns in the CANTAB implementation [114]). The participant is tasked with finding and indicating the initial sample pattern in the selection. All patterns in the selection stage are different but share a number of overlapping features. Difficulty of the task is defined by the proportion of overlapping features between the patterns – the more features are the same (overlap) across all patterns, the more difficult it is to identify the original pattern and hence the more difficult the task is. Participant accuracy at finding the original sample, measured across multiple trials, is considered to index working memory capacity [115, 116].

**Fig. 2:**
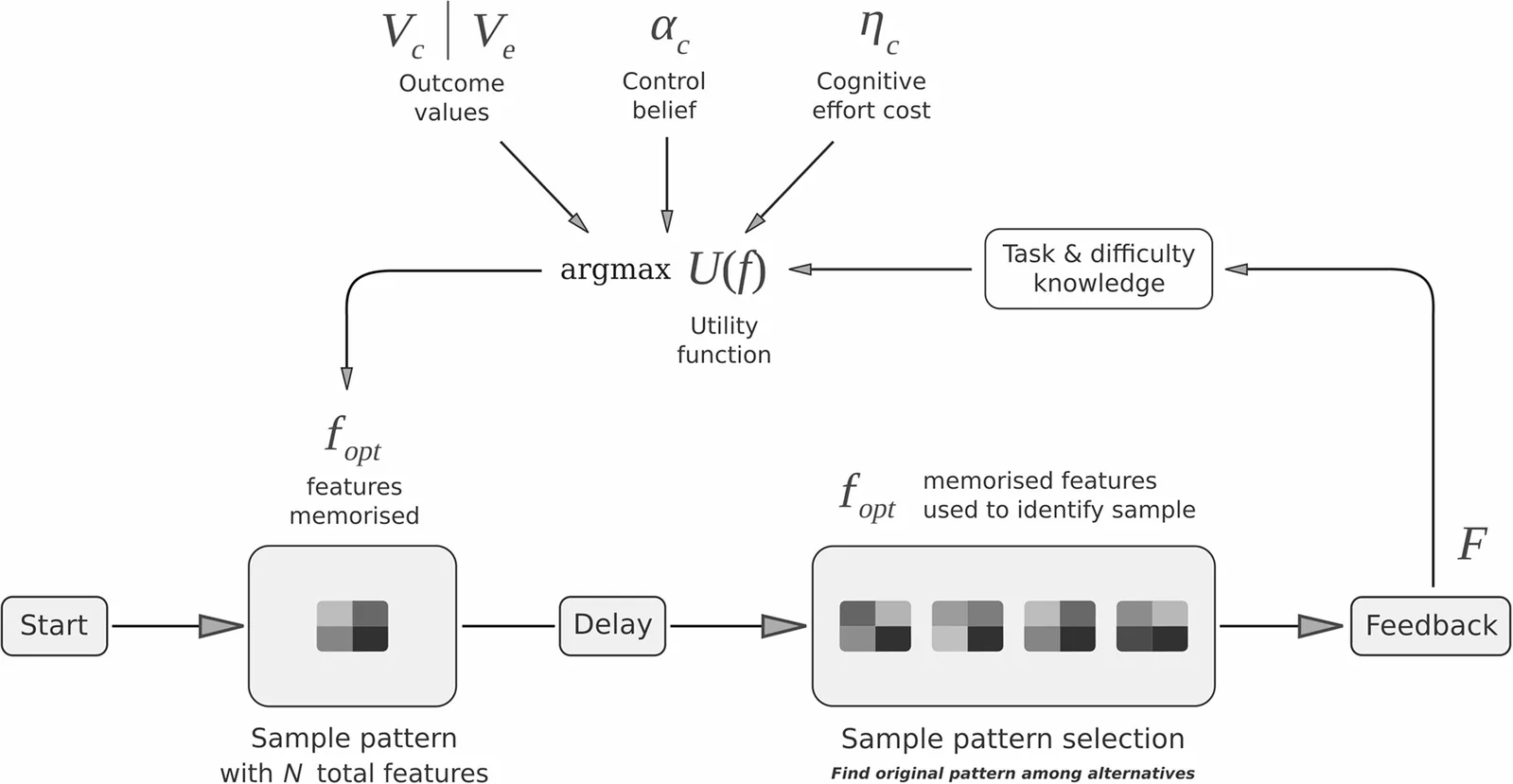
Delayed Match-to-Sample task illustration. Participant is asked to memorise a sample pattern and then to recognise that pattern in a selection after a short delay. They use their knowledge of the task, values of correct and error outcomes ($\:{V}_{c}$ and $\:{V}_{e}$), belief of control (*a*_*c*_), and cost of cognitive effort (*η*_*c*_) to estimate the number of pattern features *f* to memorise for the best reward-effort trade-off (highest utility *U*). They then implicitly memorise *f*_*opt*_ features and use them to find a matching sample at the selection stage. *f*_*opt*_ directly affects the probability of correct response. The received trial feedback *F* (correct or error) is then used to update the estimate of the task difficulty, which is used to re-estimate *f*_*opt*_ for the subsequent trial. *Note*: Patterns are for illustration only and do not represent actual stimuli typically used at the task

**Fig. 3:**
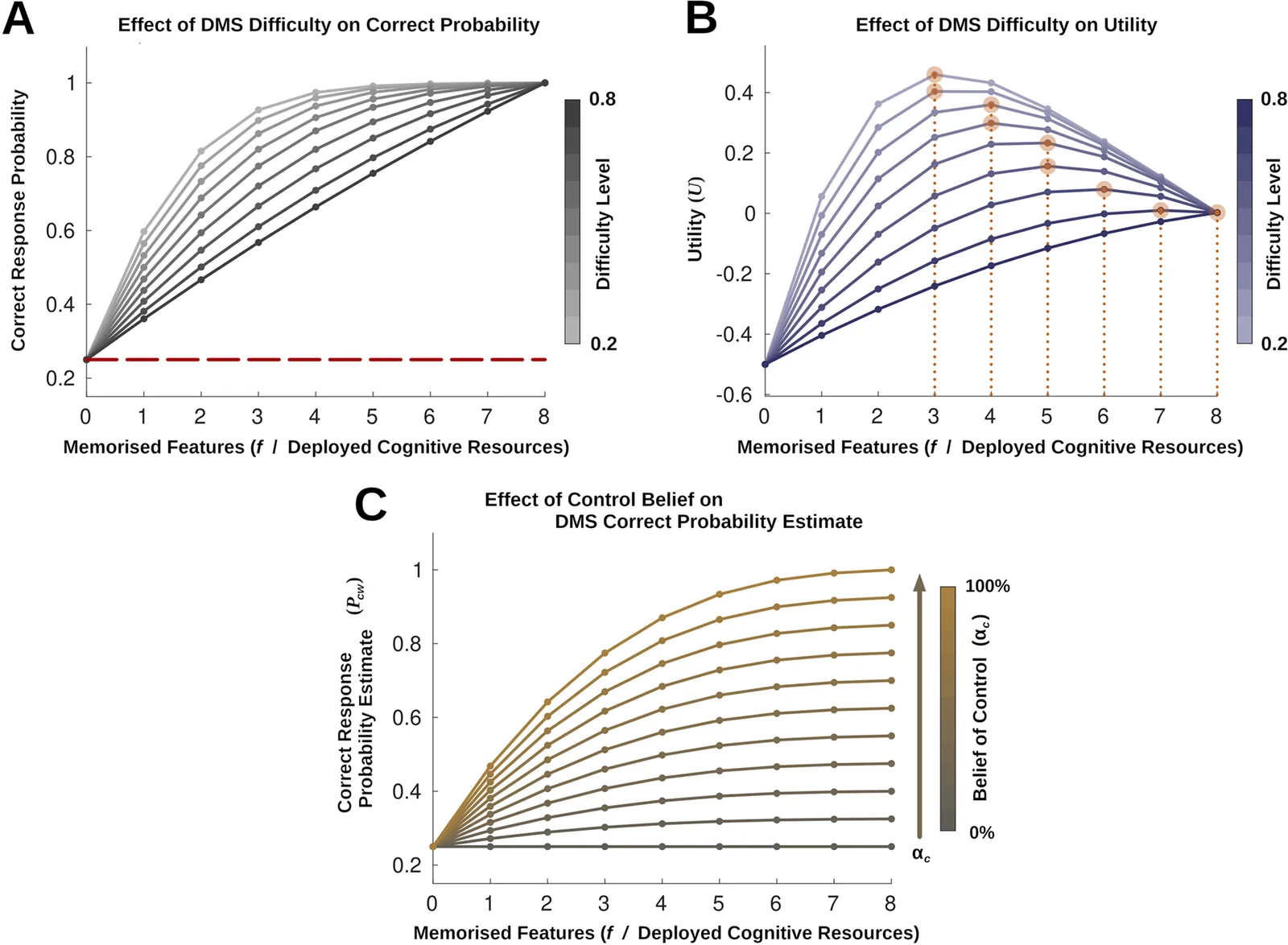
**(A)** Effect of the DMS task difficulty on the probability of correct response. Difficulty level is defined by the proportion of features which overlap (are the same) across all patterns at the selection stage – the higher the proportion, the more features the participant needs to memorise to correctly identify the original sample. When participant does not memorise any features, they perform at chance level (denoted with the red dashed line). **(B)** Effect of the DMS task difficulty on optimal utility. Orange circles with dotted lines indicate optimal utility (highest * U*, *y* axis) for different levels of difficulty (blue shades), and the corresponding numbers of features to be memorised (* f*, *x* axis). Higher task difficulty (darker line shades) leads to higher optimal number of memorised features. After errors, participants re-estimate task difficulty to be higher, and consequently memorise more features at the next trials; this leads to post-error improvement in accuracy (PIA effect). **(C)** Effect of the belief of control on participant’s estimate of the probability of correct response. Higher belief of control ($\:{\alpha\:}_{c}$, coloured grey to orange) leads to higher causal association between the number of memorised features (* f*, *x* axis) and the estimated probability of correct response ($\:{P}_{cw}$, *y* axis) – i.e., higher numbers of memorised features are estimated to lead to better chances of success (correct response)

We consider that the number of memorised features in the original sample (up to 8 features in our model, consistent with CANTAB) is directly proportional to the amount of the deployed cognitive resources, and is thus subject to the reward-effort based optimisation (* f* in Eq. 1 above). We have analytically derived the probability function for correct response dependent on the number of memorised features * f*. We further defined the participant’s belief of control ($\:{\alpha\:}_{c}$) as their estimate of the probability that their actions (the number of memorised features) and outcomes (probability of correct response) are causally associated, which is consistent with the second notion of behavioural control in Huys & Dayan (2009) [109]. We applied this definition to obtain the function of correct response probability weighted by the control belief ($\:{P}_{cw}\left(f,{\alpha\:}_{c}\right)$ in Eq. 1), which represents participant’s incorporation of their belief of control in the estimation of the probability of correct response. Figure 2 illustrates the DMS task and how outcome valuation and control belief fit into performance. The influence of the belief of control ($\:{\alpha\:}_{c}$) over the estimated probability of correct response ($\:{P}_{cw}$) is illustrated in Fig. 3C. As can be seen in Fig. 3C, higher belief of control leads to higher estimated causal association between the number of memorised features (* f*) and the probability of correct response ($\:{P}_{cw}$) – i.e., each extra memorised feature leads to a steeper increase in the estimated probability of correct response. This estimated effort-outcome association in turn affects performance by influencing the number of memorised features considered to be optimal for the reward-effort trade-off (Eqs. 1–2). Task difficulty (i.e., the proportion of overlapping pattern features) was considered to be unknown to the participant, and re-estimated with feedback after each trial. The effect of the DMS task difficulty on the correct probability function, and consequently on the utility function (Eq. 1) is illustrated in Fig. 3A and B. As can be noted in Fig. 3B, higher task difficulty leads to higher optimal number of features to be memorised. After errors, the simulated participants re-estimate the task difficulty to be higher, and consequently memorise more features in the following trials. This serves as the basis for post-error performance adaptation and the PIA effect. We compute the PIA effect in a similar way as has been done in empirical studies – as the difference between the post-error accuracy and the post-correct accuracy [42, 46]. Please see supplementary section S1.6 for further details. Please see supplementary section S1 for a complete mathematical description of the DMS task model.

We specified parameters of the DMS model to be broadly consistent with the CANTAB implementation of the task, and set valuation and control parameters of healthy controls to replicate a 90% accuracy [33, 35] and a 5% PIA effect (which is within the range for healthy participants [35]). Difficulty of the task (i.e., the proportion of features overlapping between patterns) was set to the value of $\:\frac{1}{2}$. Model performance was obtained by simulating 200 independent model runs with 1000 trials in each run (corresponding to 200 simulated participants). The number of model runs was chosen to match the number of participants considered to give sufficient power in cognitive psychological studies of group effects [117], although this is higher than the participant numbers in most previous depression studies with the DMS task [12, 90]. Please see supplementary section S1.8 for the exact details of the DMS model parameter constraints and specification.

### Eriksen Flanker Model

EF task is a two-alternative forced-choice perceptual decision-making task. At each trial, the participant is presented with a target stimulus which defines the correct response (e.g., arrows ‘<’ or ‘>’ in the arrow version of the task, indicating respectively left or right button presses). The target stimulus is presented with distractors at each side (flankers), which are either congruent with the target and indicate the same response, or incongruent and indicate a conflicting response [118]. In the arrow version of the task, a congruent trial consists of five arrows with the same direction (e.g., ‘< < < < <’). Incongruent trials have target and flanker stimuli aimed in different directions (e.g., ‘> > < > >’). Difficulty of the task is defined by the proportion of incongruent trials – the more incongruent trials there are, the more attention the participant needs to pay to the target stimulus to remain accurate. Reaction times and error rates at the task are considered to index selective attention and inhibitory cognitive control.

**Fig. 4:**
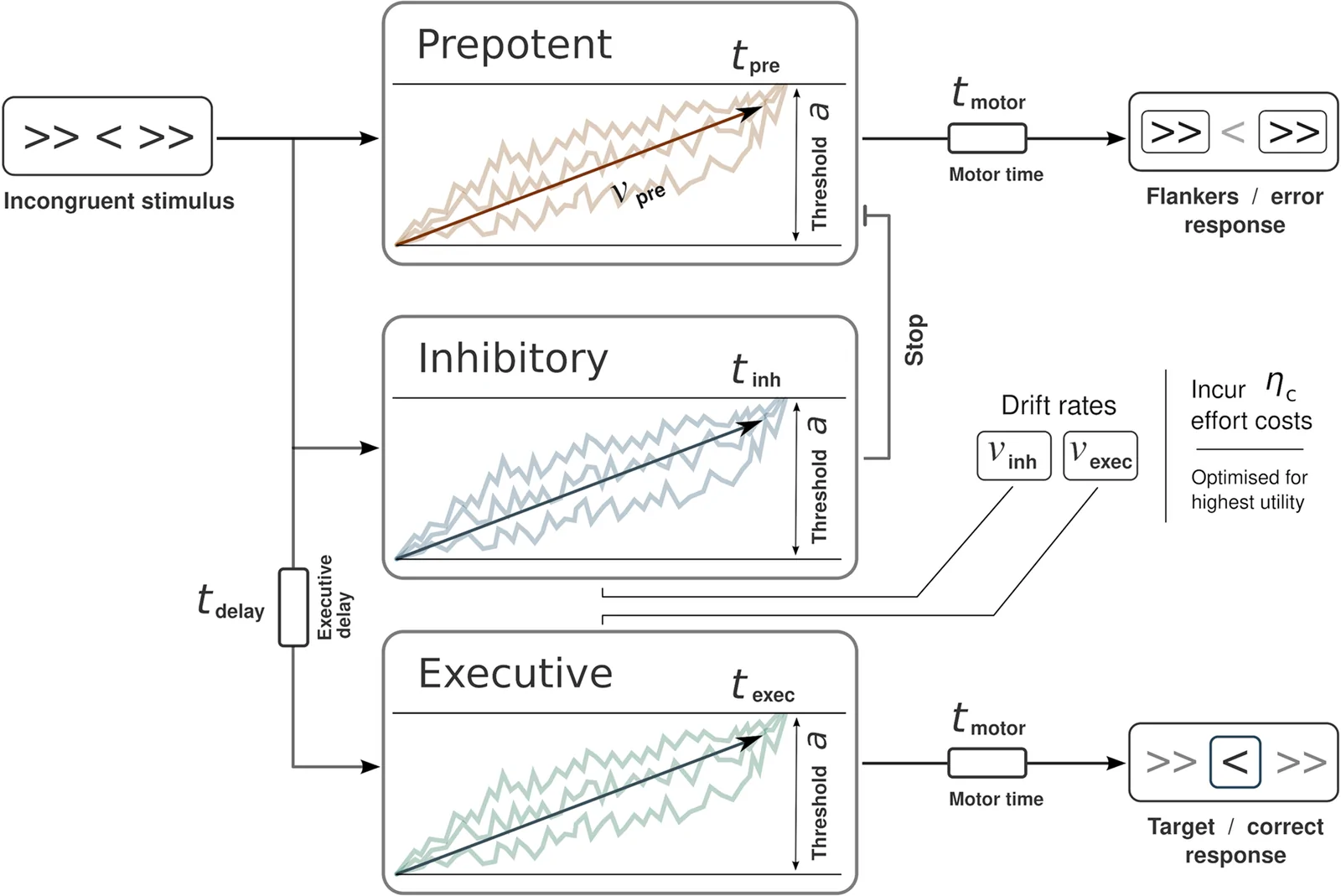
Eriksen Flanker task drift-diffusion model framework [28, 119]. *Prepotent* process leads to error responses at incongruent trials. When the *prepotent* process is stopped by the *inhibitory control* process, *executive control* process is able to complete a correct response. Inhibitory and executive processes are effortful and their respective drift rates ($\:{v}_{inh}$ and $\:{v}_{exec}$) incur the cognitive effort costs $\:{\eta\:}_{c}$. These two drift rates are optimised to maximise the reward-effort trade-off (similarly to the number of features * f* in the DMS task)

To model reaction times and error rates at the task we applied the drift-diffusion framework (DDM) of Dillon et al. (2015) [28], which was in turn adapted from Noorani & Carpenter (2013) [119]. This framework is illustrated in Fig. 4 and further details are provided in supplementary section S2.1. Briefly, the framework consists of three drift-diffusion processes: prepotent process which defines response times at congruent trials, inhibitory process which prevents errors at incongruent trials, and executive process which defines response times at incongruent trials. Inhibitory and executive processes are related to goal-driven cognitive control and are effortful – the more cognitive resources are allocated to these processes, the faster and more accurate is task performance. We consider that cognitive effort allocated at the EF task is proportional to the drift rates of the inhibitory and executive processes ($\:{v}_{inh}$ and $\:{v}_{exec}$ in Fig. 4), and that they are subject to the reward-effort based optimisation similarly to the number of memorised features in the DMS task (i.e., equivalent to * f* in Eq. 1). Drift rates in DDM are considered to represent the neural activity related to evidence accumulation [120], which is costly from the neurometabolic perspective [121]. We thus consider these rates to incur the effort costs in the model ($\:{\eta\:}_{c}$ in Eq. 1). We analytically derived the probability function for correct response dependent on the inhibitory drift rate, and applied our definition of control belief ($\:{\alpha\:}_{c}$) to obtain the function of correct response probability weighted by the control belief ($\:{P}_{cw}\left(f,{\alpha\:}_{c}\right)$), similarly to the DMS task. As with the DMS task, control belief ($\:{\alpha\:}_{c}$) in the EF task model corresponds to the simulated participant’s estimate of the general probability of causal association between their effort (inhibitory drift rate $\:{v}_{inh}$) and the trial outcome (probability of correct response). Please see supplementary section S2 for a detailed description of our model of the EF task. We opted to use the DDM of Dillon et al. (2015) because it was already constrained and parametrised to match the behavioural performance of control participants, and because it can be directly linked to the EVC theory when the inhibitory and executive drift rates are considered to be proportional to the task-allocated cognitive effort. This link is particularly important in our study because it helps address the hypothesis that changes in motivational factors in depression translate to lower cognitive effort, which is reflected in the reduced drift rates, that in turn lead to deficits in performance.

Parameters of the EF DDM (Fig. 4) were specified to closely approximate the values derived for control participants in Dillon et al. (2015) [28], with the exception for the inhibitory and executive drift rates because these are considered to be optimised to maximise the reward-effort trade-off. Difficulty of the task (proportion of incongruent trials) was set to $\:\frac{1}{2}$. Similarly to the DMS model, performance measures were obtained by simulating 200 independent model runs with 1000 trials in each run (corresponding to 200 simulated participants). Please see supplementary section S2.7 for the exact details of the EF model parameter specification. Table 1 summarises the modelled EF performance measures compared to the experimental results of Dillon et al. (2015) [28].

**Table 1:** Eriksen Flanker task control model performance

	Experimental	Modelled
*Congruent RT*	360 ms	367 ms
*Incongruent* *Correct RT*	448 ms	448.5 ms
*Incongruent* *Error RT*	319 ms	328.4 ms
*Incongruent* *Correct Rate*	76.2%	73.3%
PIA	1%	5.07%
ERN	-	1.064

Note: ERN is in abstract units related to the valuation of correct and error performance (Eq. 3).

### Depression Modelling

As described above, we first specified and parametrised the DMS and EF models to replicate performance of healthy control participants [28, 33, 35]. We then introduced depression-related motivational factors, as we describe below, to check if they can lead to the cognitive performance deficits. Within the DMS model, we aimed to find affective parameter alterations which can result in decreased accuracy and decreased or eliminated PIA effect [12, 33–35]. Within the EF model, we aimed to replicate increased correct response times at incongruent trials [28], and to test which parameter changes might lead to decreased or increased accuracies [28, 92, 97, 98], as well as decreased or increased ERN magnitudes [92–94, 98–100]. Following the aims of the study, we constrained investigations of depression to the following three possibilities: (1) a decrease in the $\:{\alpha\:}_{c}$ parameter (control belief deficit, representing *learned helplessness*); (2) a decrease in the $\:{V}_{c}$ parameter (representing anhedonic decrease in reward sensitivity); (3) an increase in the magnitude of the $\:{V}_{e}$ parameter (representing biased negative valuation, note that $\:{V}_{e}$ is always negative).

## Results

### Delayed Match to Sample Modelling Results

**Fig. 5:**
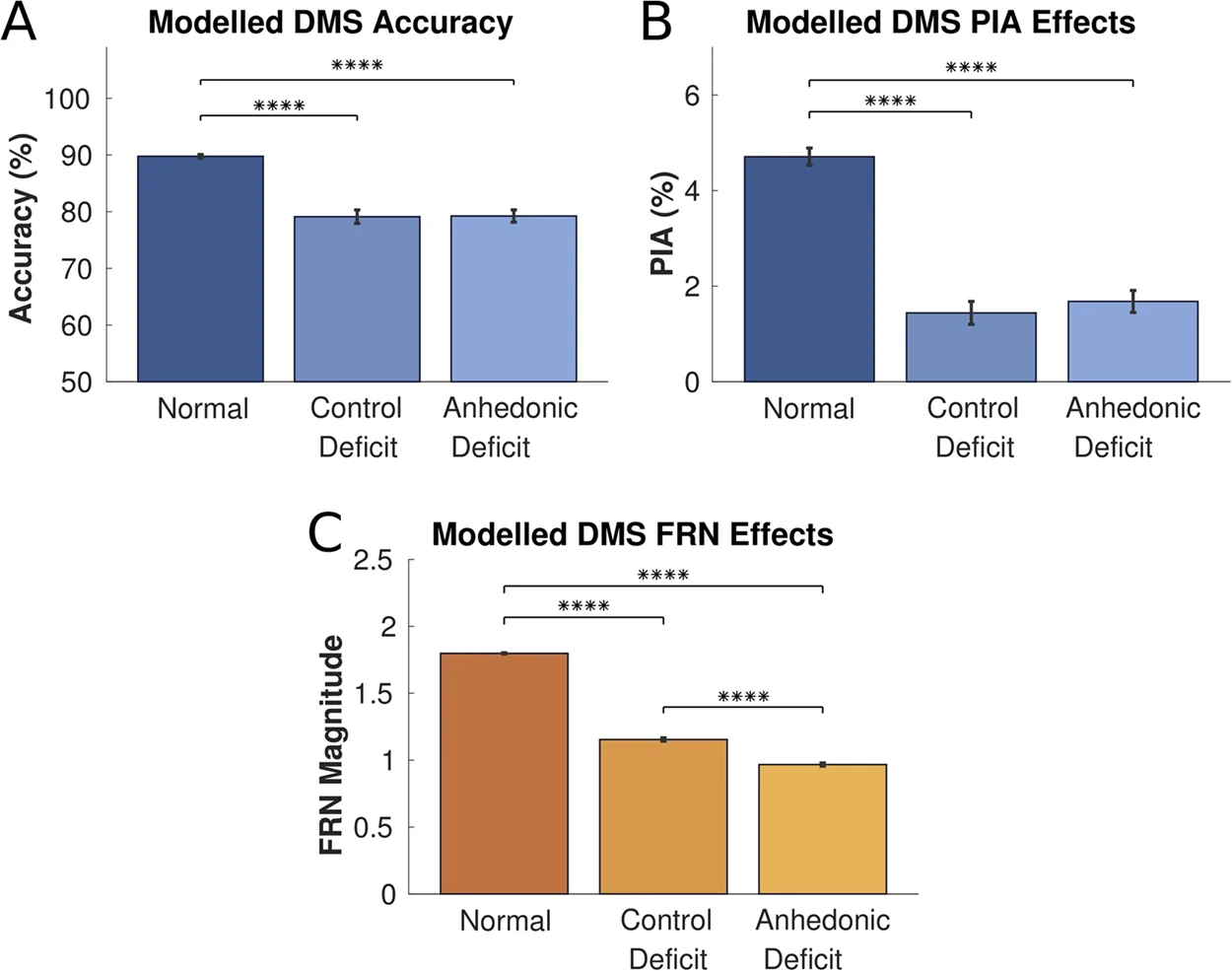
Modelled depression effects over DMS measures: (**A**) Performance accuracy; (**B**) Post-error accuracy improvement (PIA) effect; (**C**) FRN magnitude. Four stars indicate significant differences (*p* < 0.0001). Error bars in plots A and C represent standard deviations of the mean. Error bars in plot B represent standard errors of the mean. Control belief and anhedonic valuation deficits result in similar decreases in accuracy and the PIA effect (A and B) but have different effects on the FRN magnitude (C)

Simulation results revealed that two out of the three investigated depression-related deficits could replicate decreased accuracy and decreased PIA effect within the DMS model. Performance deficits were replicated by either the decreased control estimate ($\:{\alpha\:}_{c}$) or the decreased reward value ($\:{V}_{c}$), but not by an increase in the negative outcome value ($\:{V}_{e}$). These results held across different explored decreased values in $\:{\alpha\:}_{c}$ and $\:{V}_{c}$ parameters. To compare the two models of depression between each other, we constrained them to produce an approximate 10% decrease in accuracy – this corresponded to a 79% decrease in reward value and a 40% decrease in control belief (supplementary section S1.9). Results of the model simulations can be seen in Table 2, Fig. 5. Simulations predicted a higher decrease in the FRN for the anhedonic deficit as compared to the control belief deficit (*P *<* 0.00001*), with otherwise similar decreases in accuracy and the PIA effect (*P *>* 0.10*). 

**Table 2:** Delayed match to sample modelled control and depression measures

	*Accuracy*	*Post-Error* *Error Rate*	*Post-Correct* *Error Rate*	*PIA*	*FRN*
Control	89.77%	6%	10.71%	4.71%	1.80
Reward Sensitivity Deficit	79.23%	19.41%	21.09%	1.68%	0.97
Control Belief Deficit	79.11%	19.72%	21.16%	1.44%	1.15

Note: FRN is in abstract units related to the valuation of correct and error performance (Eq. 3).

### Eriksen Flanker Modelling Results

Simulation results showed that all three depression-related deficits led to increased incongruent correct response times, which again held across different explored parameter values. To compare the three depression models, we constrained them to approximate the mean incongruent correct response time of depressed participants in Dillon et al. (2015) (approx. 475 ms in depression vs. approx. 450 ms control) [28]. To investigate if the different depression-related factors might have counteracting effects when they co-occur (i.e., are present together), we simulated two additional combinations – control belief deficit together with negative value deficit, and reward sensitivity deficit with negative value deficit. Table 3 illustrates qualitative effects of the depression models over task performance (please see supplementary section S2.8 for the exact EF depression model parameter values and the exact effects of depression models over performance). Control belief and reward sensitivity deficits resulted in similar qualitative patterns – increased RT for incongruent trials (both error and correct), decreased incongruent trial accuracy, and decreased ERN magnitude. No change in the PIA effect was observed with these models. The aversive deficit model resulted in an opposite pattern for all measures except the incongruent correct RT. The two additional combined models resulted in spared performance on all measures except for the incongruent correct RT and the ERN, both of which were increased. This suggests that when some of the motivational deficits co-occur, they may have counteracting effects on cognitive performance.

**Table 3:**
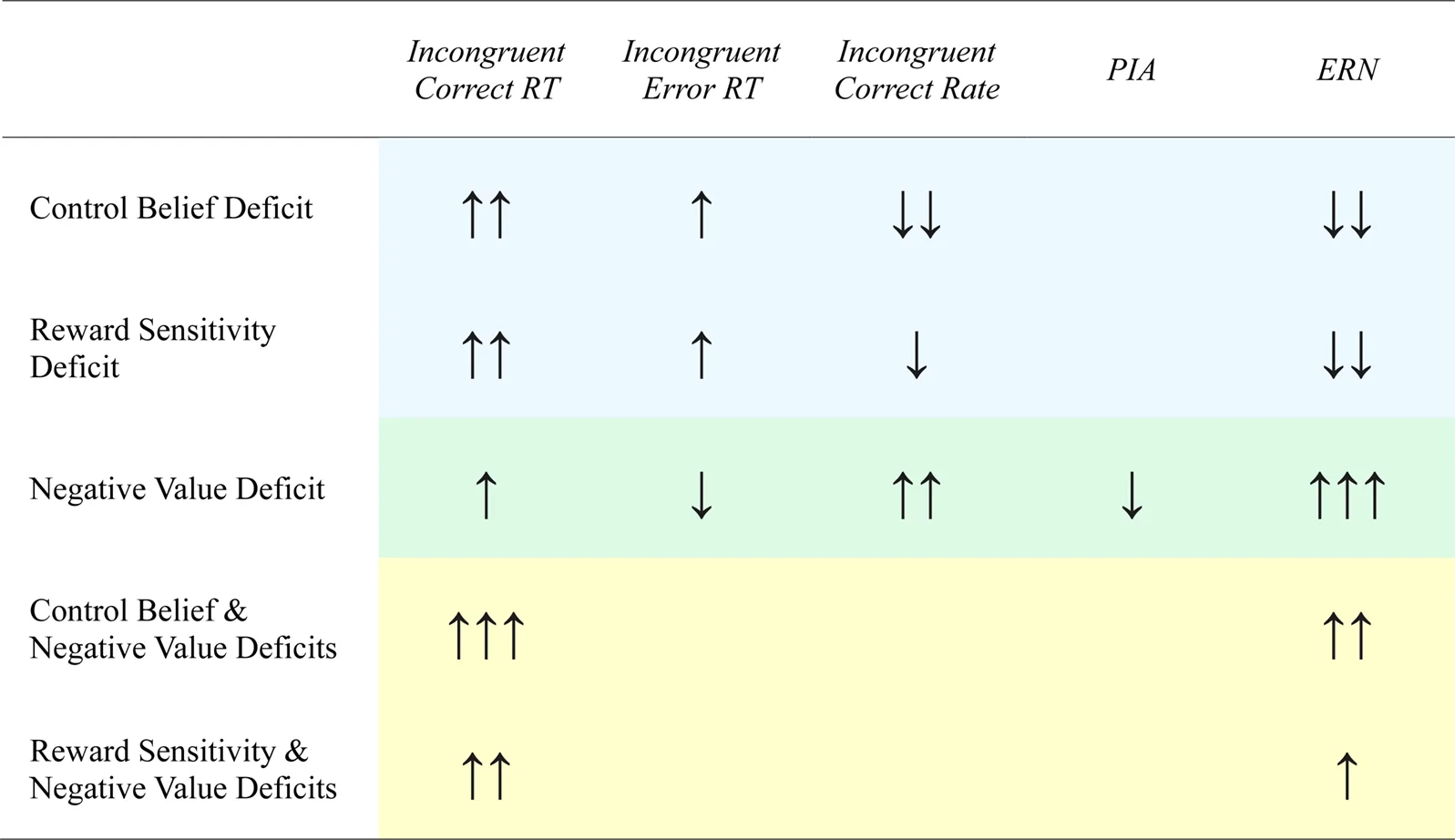
Qualitative depression model effects over EF task performance

Note: Similar qualitative performance patterns are highlighted with similar colours

## Discussion

### Summary of Results

We constructed two computational models which explicitly link motivational factors (outcome valuation, belief of control) to cognitive performance at the DMS and EF tasks. The models are based on the EVC theory, which posits that the brain allocates cognitive resources in a way so as to maximise reward-effort trade-off [81–83]. We further introduced depression-related motivational factors (anhedonic reward sensitivity, negative valuation and perceived lack of control) in the models to see if they can account for selected performance deficits in depression [12, 28, 33–35, 92–94, 97–100]. Results revealed that lower belief of control (“learned helplessness”) and anhedonic reward sensitivity could account for reduced accuracy, PIA effect and FRN potential at the DMS task, with reduced reward sensitivity leading to higher FRN decrease. At the EF task, different depression-related motivational factors led to different performance patterns, but all resulted in increased correct response times at incongruent trials (Table 3). Overall, the results show that depression-related motivational factors can causally account for cognitive performance deficits, and that patterns of cognitive performance could potentially serve as a basis for stratifying depression according to motivational changes. These results are interesting because they suggest that cognitive deficits may not necessarily always be due to mechanistic changes in the brain [122], and that treating core affective or motivation-related aspects of depression could potentially also ameliorate cognitive deficits at least in some depression subtypes.

### Depression Modelling

Depression-related cognitive deficits within our models arise because of changes in utility estimation. Specifically, altered control belief and outcome valuation lead to higher utility being assigned to the deployment of lower amounts of cognitive resources, and subsequently to poorer performance due to less resources deployed (Eqs. 1 and 2, Fig. 1). As such, our models only provide a normative (rational) explanation of the aetiology of cognitive deficits, but do not consider that they may arise as a consequence of other cognitive or mechanistic changes in the brain, which is possible in more severe depression subtypes [11, 103, 108]. One additional normative explanation of cognitive performance deficits could be in an increase in the costs of cognitive effort ($\:{\eta\:}_{c}$ in Eq. 1). We limited the scope of this study to focus on the changes in outcome valuation and control belief because they are linked to prominent theories of depression and supported by historic literature [71–76, 80]. However, emergent evidence does indicate that costs of effort are likely also altered in depression [123, 124], and we acknowledge that this should be investigated as a possible alternative or additional normative substrate of cognitive deficits in future studies [84]. In addition to this, we here did not consider that poorer cognitive performance could arise due to disruption of working memory or attentional task-sets brought on by task-irrelevant interfering cognitive or emotional processes such as long-term memory retrieval or rumination [9]. These remain plausible alternative mechanisms of cognitive deficits that would correspond to depression subtypes other than those considered in the current study.

### Modelling Predictions

Apart from causally linking motivational factors to cognitive performance, our model of the DMS task made a specific prediction about the magnitude of the FRN signal [110–113]. Specifically, the anhedonic reward sensitivity model of depression predicted a larger decrease of the FRN signal compared to the control belief deficit, with otherwise comparable decreases in accuracy and the PIA effect (Fig. 5). This prediction could be directly tested experimentally by evaluating a group of depressed participants with higher anhedonia scores and lower helplessness scores against a group with higher helplessness and lower anhedonia scores. The former group should display lower FRN magnitudes compared to the latter, when both groups are matched for the DMS task accuracy.

Regarding the EF task, our simulations revealed three distinct qualitative patterns of depression-related performance (Table 3), which supports the notion that depression may be stratified on the basis of performance at cognitive tasks. Decreased accuracy and decreased ERN signal were attributed to lower control belief or reward sensitivity – thus, in future empirical studies, these measures could be hypothesized as indicative of responsiveness to behavioural activation therapy [125], or to dopaminergic medication [126, 127]. Increased accuracy and increased ERN signal, on the other hand, were related to increased negative valuation, and thus future empirical studies could aim to test whether these measures may be indicative of responsiveness to anxiolytic treatments such as higher doses of SSRI or cognitive behavioural therapy [128–130]. The empirical findings of the ERN signal change in depression are mixed [63–66] – our results directly address this from a theoretical perspective and suggest that directly opposite changes in this signal could be due to the different predominant motivational changes.

Although our models offer a useful conceptual, theoretical and computational bridge between motivation and cognitive deficits in depression, they are not yet clinically applicable at the current stage. One way in which the models could be moved closer to clinical translation is through further development towards inference of motivation-related parameters from behavioural data [131]. Patient’s control and value parameters estimated in this way, together with other data, could potentially help refine the diagnosis and select an optimal treatment, as we briefly discuss above [132, 133]. However, optimal treatment selection in real-world clinical practice is complex, with decisions usually based on multiple factors, including individual symptoms, severity and co-morbid conditions [134]. Future work should (1) evaluate and ensure that the motivation-related parameters of the models are recoverable, and implement any model changes that may be needed for this (e.g., additional parameter constraints informed by extra data), (2) fit the models to individual-level or trial-by-trial data and validate that the model fits can account for core features of the data, and (3) evaluate whether the estimated motivation-related parameters are clinically relevant, taking into account complexities such depression severity and co-morbidities [135, 136].

We note that our model of the EF task was constrained with the parameter values and behavioural measures reported in Dillon et al. (2015) [28] (supplementary section 2.7), and thus testing the predictions of our model is contingent on application of the same version of the EF task as in Dillon et al. (2015). Future studies should apply the similar DDM framework to other datasets with the EF task, which would enable tailoring and validating our model for other versions of the task.

### Neurobiological Interpretation

As regards the neurobiological interpretation, the core resource allocation computation (Eqs. 1–2) is theorised to be completed in the dorsal anterior cingulate cortex (ACC) within the EVC account, which is supported by a range of human and animal studies [81–83]. Outcome values could be extracted from long-term affective memory in the amygdala and the ventral striatum upon first presentations of positive and negative feedback [137, 138], then maintained in the orbitofrontal cortex and the insula [139–142], and signalled to the ACC for resource allocation computations [81, 82, 142]. Belief of control is likely maintained and signalled by multiple areas of the prefrontal cortex, but also possibly by tonic levels of serotonin [71–73, 143]. Actual control over task execution is likely performed by specific sub-regions of the lateral prefrontal cortex (LPFC), dependent on the requirements of the task [83, 144]. Prime candidates for cognitive resources are the neurotransmitters needed to modulate neural activity in the LPFC, as well as the sources of metabolic energy for prolonged firing of neural cells. These neurotransmitters include dopamine, noradrenaline and acetylcholine [145–148], whereas the known depletable metabolic energy sources in the brain include blood glucose, adenosine triphosphate and astrocytic glycogen [83, 121, 149, 150]. How exactly the ACC may direct these neural resources for task completion remains an interesting avenue for future work. Although we modelled the simplest possible (linear) relationship between the deployed cognitive resources and cognitive costs (term $\:{\eta\:}_{c}\cdot\:f$ in Eq. 1), the exact nature of this costing computation could be more complex and non-linear, and could depend on the type of neural resources deployed and their availability. This remains an avenue for further research [83]. In general, although the above neurobiological interpretation is plausible, it remains in many parts hypothetical, and research is needed to further substantiate the EVC theory and to confirm the exact neural substrates of its different components.

### Limitations

Despite the explanatory and predictive advantages of our computational account, there are some limitations. First, we modelled optimal cognitive resource allocation in terms of explicit computations of outcome probabilities and utility (Eqs. 1–2, supplementary sections S1.2-S1.4 and S2.2-S2.4). In practice, however, it is more likely that outcome probabilities ($\:{P}_{cw}$ in Eq. 1) are either approximated or gradually learned over time and cached in the brain, to reduce computational complexity. This process could take place over the initial trials of the task and engage neural systems for reinforcement learning [151]. To focus on the effects of interest, we did not model complete statistical or reinforcement learning and instead constructed a simplified account of continuous re-estimation of task parameters (task difficulty) based on trial-by-trial feedback, which explains post-error accuracy improvement (supplementary sections S1.5-S1.6 and S2.5). In practice, learning about the task and outcome values is likely to be a more complex process, which may itself be altered in depression. Some participants, for example, may also have more complex implicit beliefs, such as the possibility that higher difficulty of the task is related to higher reward (feedback) values. We did not consider these more complex cases in our study, however they could be explored in future work with extended versions of our models. Further to these points, we only focused on the measures of working memory and attention, and did not tackle depression deficits in other domains such as learning and long-term memory [12, 14, 16, 29]. The EVC framework has recently been extended to these domains – particularly to learning [152, 153] – and further application of this framework to better explain depression-related learning and memory deficits remains an important avenue for future work. Finally, apart from depression, cognitive performance can be independently affected by demographic and clinical factors such as age, biological sex, or clinical conditions that can impact brain health. These factors will need to be taken into account or controlled for when testing the model predictions, or when fitting the models to individual-level data, in future studies. Further to that, it is possible that variability in motivation-related parameters may be driven by the severity of depression – this will also need to be tested in future model-fitting studies.

## Conclusion

Depression remains a prevalent and impactful condition which adversely affects cognitive function. In the current study we showed that, from a theoretical perspective, deficits in working memory and attention at the DMS and EF tasks could be causally accounted for by the depression-related motivational factors – decreased belief of control and altered valuation of outcomes. Computational modelling also made several predictions which could be tested experimentally by carefully evaluating cognitive performance measures against the predominant motivational symptoms. Taken together, these results can help form new hypotheses related to depression stratification, and possibly selection of optimal treatments, in the future empirical studies.

## Supplementary Information

Below is the link to the electronic supplementary material.

Supplementary Material (PDF, 304 KB)

## Data Availability

No datasets were generated or analysed in the current study.
